# Supplementary material for: Iron Hydroxide/Oxide-Reduced Graphene Oxide Nanocomposite for Dual-Modality Photodynamic and Photothermal Therapy In Vitro and In Vivo
Source: Nanomaterials (Basel). 2021 Jul 28;11(8):1947. doi: 10.3390/nano11081947 (PMC8402170; doi:10.3390/nano11081947)
Supplement: Supplementary file 1 [file nanomaterials-11-01947-s001.zip › nanomaterials-1292474-supplementary.pdf]

nanomaterials

Supplementary Materials

Iron Hydroxide/Oxide-Reduced Graphene Oxide Nanocomposite for Dual-Modality Photodynamic and Photothermal Therapy In Vitro and In Vivo

Wei-Jane Chiu [1], Yi-Chun Chen [2], Chih-Ching Huang [1,3,4], Lingyan Yang [5], Jiantao Yu [5], Shih-Wei Huang [6,7,8] and Chia-Hua Lin [2,*]

S1. Photothermal Conversion Efficiency of FeOxH–rGO

The photothermal conversion efficiency of FeO_xH–rGO was determined as previously described [1]. The temperature change of the aqueous dispersion of FeO_xH–rGO was recorded as a function of time under continuous irradiation of NIR 808 nm with a power intensity of 2.72 W/cm^2. The real-time temperature was recorded every 10 s. The laser was shut off when the aqueous dispersion reached the highest temperature. To determine the rate of heat transfer from the dispersion system to the environment, the temperature decrease of the aqueous dispersion was continuously monitored. We calculated the photothermal conversion efficiency (η) using the following equation:

$$\eta = \frac{hS(T_{\max} - T_{sur}) - Q_{dis}}{I(1 - 10^{-A_{808}})} \cdots\cdots (1)$$

where h is the heat transfer coefficient, S is the surface area of the container, T_{max} is the equilibrium temperature and T_{sur} is the surrounding temperature. The Q_{dis} represents the baseline energy input by the system, I is the intensity of the laser input and A_{808} is the absorbance of FeO_xH–rGO at the wavelength of 808nm. The photothermal conversion efficiency of FeO_xH–rGO was estimated to be 64.4%.

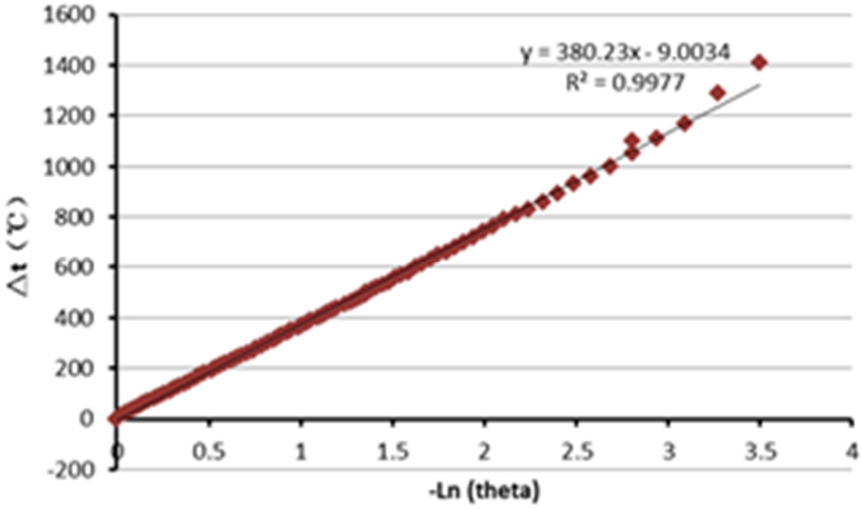

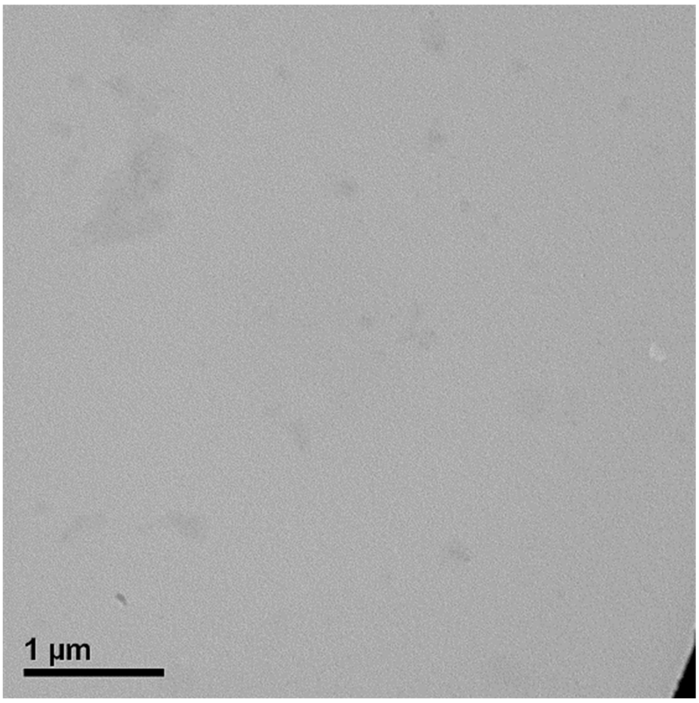

Figure S1. TEM images of rGO. rGO (size ~300 nm) was prepared from irradiation of GO with UV light for 5 h.

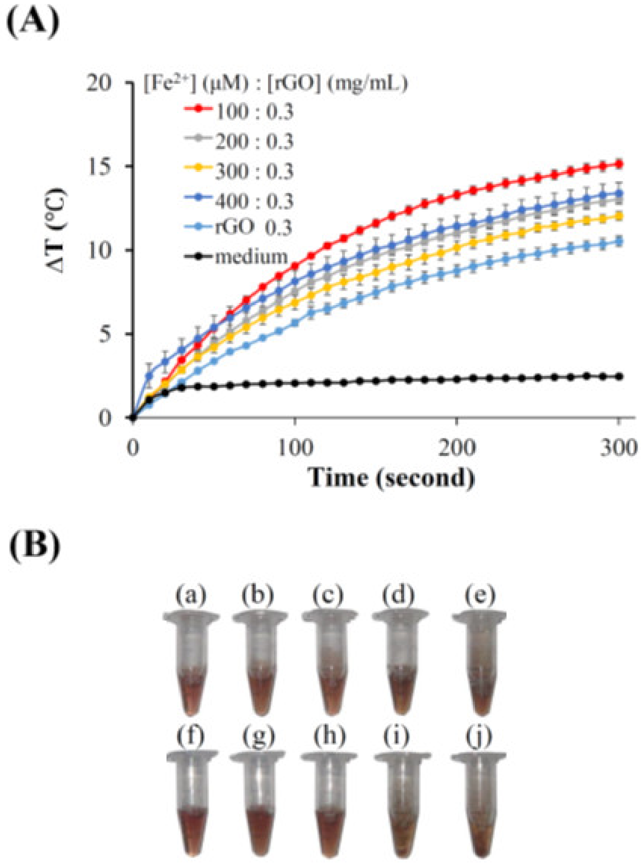

Figure S2. (**A**) Differential temperature (ΔT) profiles as a function of irradiation time for pure medium, rGO (0.3 mg/mL) and above FeO_xH–rGO nanocomposites solutions in medium, respectively, were irradiation by an 808 nm NIR (1.82 W/cm^2) for 5 min, and (**B**) photos of FeO_xH–rGO nanocomposites prepared by 100, 200, 300 and 400 µM of Fe^{2+} ions reacted with rGO (300 µg/mL) in medium before (a–e) and after (f–j) NIR irradiation (808 nm, 1.82 W/cm^2) for 5 min.

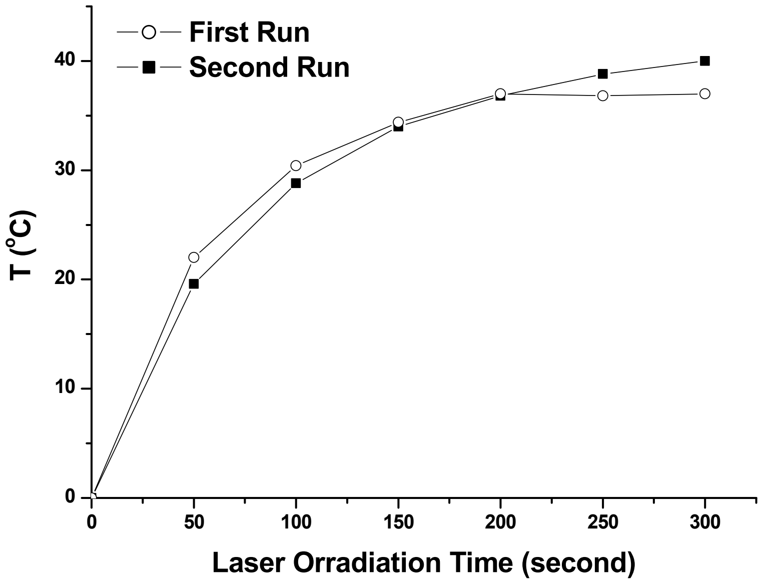

Figure S3. Photothermal stability of FeOxH–rGO nanocomposites (300 μg/mL) under NIR illumination.

Reference

1. Roper, D. K.; Ahn, W.; Hoepfner, M., Microscale heat transfer transduced by surface plasmon resonant gold nanoparticles. *J Phys Chem C* **2007,** *111*, (9), 3636-3641.
